# Comparison of MR cytometry methods in predicting immunohistochemical factor status and molecular subtypes of breast cancer

**DOI:** 10.2478/raon-2025-0044

**Published:** 2025-08-06

**Authors:** Lei Wu, Fan Liu, Sisi Li, Xinyi Luo, Yishi Wang, Wen Zhong, Thorsten Feiweier, Junzhong Xu, Haihua Bao, Diwei Shi, Hua Guo

**Affiliations:** Qinghai University Affiliated Hospital, Xining, China; Center for Biomedical Imaging Research, School of Biomedical Engineering, Tsinghua University, Beijing, China; MR Research Collaboration Team, Siemens Healthineers Ltd., Beijing, China; Tsinghua Shenzhen International Graduate School, Tsinghua University, Shenzhen, China; MR Research Collaboration Team, Siemens Healthineers AG, Erlangen, Germany; Institute of Imaging Science, Vanderbilt University Medical Center, Nashville, TN, United States; Department of Biomedical Engineering, Vanderbilt University, Nashville, TN, United States; Department of Radiology and Radiological Sciences, Vanderbilt University Medical Center, Nashville, TN, United States; Center for Nano and Micro Mechanics, Department of Engineering Mechanics, Tsinghua University, Beijing, China

**Keywords:** MR cytometry, diffusion MRI, OGSE, breast cancer subtyping, transcytolemmal water exchange

## Abstract

**Background:**

First evaluation of the performance of MR cytometry incorporating transcytolemmal water exchange in predicting immunohistochemical factor status and molecular subtypes of breast cancer.

**Patients and methods:**

We prospectively enrolled 90 breast cancer patients in the study. For each participant, pulsed gradient spin-echo (PGSE) with diffusion time of 70 ms and oscillating gradient spin-echo (OGSE) diffusion-weighted imaging of 25 Hz and 50 Hz were performed on a 3T MRI scanner. Time-dependent apparent diffusion coefficients (ADC) and microstructural parameters including cell diameter ***d***, intracellular volume fraction ***v_in_***, water exchange rate constant ***k_in_***, and apparent extracellular diffusivity ***D_ex_*** were calculated. Single- and multi-variable logistic regression analyses were performed to evaluate their performance in identifying immunohistochemistry (IHC) factor status and molecular subtypes. The area under the receiver operating characteristic curve (AUC) was computed.

**Results:**

The multi-variable regression models generated from MR cytometry-derived metrics provided higher AUC compared to those from time-dependent ADC metrics, *i.e*. 0.744 *vs*. 0.645 for estrogen receptor (ER), 0.727 *vs*. 0.688 for progesterone receptor (PR), 0.734 *vs*.0.623 for HER2, and 0.679 *vs*. 0.633 for Ki67, 0.751 *vs*. 0.644 for Triple-Negative Breast Cancer (TNBC), 0.819 *vs*. 0.765 for HER2-enriched, 0.730 *vs*. 0.659 for Luminal A, 0.633 *vs*. 0.633 for Luminal B. MR cytometry with transcytolemmal water exchange (JOINT and EXCHANGE) outperformed the original one with the impermeable model (IMPULSED) in predicting PR (0.727 *vs*. 0.705), HER2 (0.734 *vs*. 0.689), Ki67 (0.679 *vs*. 0.646), TNBC (0.751 *vs*. 0.748) and HER2-enriched (0.819 *vs*. 0.739), Luminal A (0.730 *vs*. 0.666), Luminal B (0.633 *vs*. 0.630).

**Conclusions:**

MR cytometry outperformed conventional ADC measurements in clinical breast cancer subtyping. Incorporating transcytolemmal water exchange further enhanced classification accuracy.

## Introduction

Breast cancer stands as one of the most prevalent malignant diseases affecting women, with its incidence and mortality rates rising annually.^1^ There are significant differences in terms of malignancy, therapeutic strategies and prognosis across breast cancers with different molecular subtypes.^2–4^ Accurate subtype identification is crucial for developing personalized treatment plans for individual patients to reduce mortality and improve prognosis.^5^ Currently, the categorization of breast cancers usually depends on multiple biomarkers from pathological immunohistochemistry (IHC) examinations, and the corresponding subtypes are primarily determined by invasive biopsies with the risks of oedema, bleeding, and infection.^6^ Furthermore, biopsy may lead to missed detection due to the limitation of a localized pathological puncture point, which cannot reflect the overall situation of the lesion.^7^

Imaging techniques that allow for both non-invasive and wide-area detection provide various promising approaches for the diagnosis of breast cancer.^8^ Among them, MRI has been widely used in breast imaging, with its advantages of high image resolution, no ionizing radiation, and multi-contrast imaging, which greatly compensates for the limitations of biopsies and provides more comprehensive information.^9^ Diffusion MRI (dMRI) can reveal tumor microstructures by non-invasively probing the diffusion movement of water molecules without any exogenous contrast agents. The dMRI-derived metric, apparent diffusion coefficient (ADC), has been widely used in the clinical diagnosis^10^ and posttreatment evaluation^11^ of breast cancers. However, this metric represents non-specific, averaged information influenced by several microstructural features with competing effects, reducing diagnostic sensitivity.^12^ A meta-analysis^13^ has shown that the ADC values significantly overlapped among different breast cancer subtypes.

Current ADC measurements in clinics usually adopt a single diffusion time *t_diff_*. Over the last decade, there has been an increasing interest in obtaining ADCs with different *t_diff_*. Because the measurement of water diffusion is dependent on *t_diff_*, ADCs with varying probe varying length scales.^14^ Therefore, multiple *t_diff_*-based ADC provide more comprehensive information on tissue microstructure. Such a technique is usually termed time-dependent diffusion MRI (t_d_-dMRI), which has been widely used in cancer imaging.^15,16^ However, time-dependent ADCs still represent the averaged diffusion behavior so it suffers from low specificity of tissue properties. The recently emerging technique of MR cytometry imaging^17–19^ provides a potential approach to address the above limitation, which implements multiple-*b* and multiple-*t_diff_* acquisitions, combined with multi-compartmental biophysical modeling, to decouple different microstructural parameters, such as cell diameter *d*, intracellular volume fraction *v_in_*, and apparent extracellular diffusivity *D_ex_*. The Imaging Microstructural Parameters Using Limited Spectrally Edited Diffusion (IMPULSED)^17^, a specific form of MR cytometry, incorporates both the pulsed-gradient spin-echo (PGSE) and oscillating gradient spin-echo (OGSE)^20^ acquisitions for a much broader diffusion time range for more comprehensive length scale information, and more importantly, it is readily achievable on 3T MRI scanners with a clinically-feasible scan time of ~6 minutes.^17^ For this reason, IMPULSED has been implemented in breast cancer^17,21,22^, prostate cancer^23^ and gliomas.^24^

Wang *et al*.^22^ have validated the diagnostic efficacy of IMPULSED^17^ to classify molecular subtypes of breast cancer. Despite the successful applications, IMPULSED assumes tumor cells as impermeable spheres (without transcytolemmal water exchange), so that dMRI signals from intra-and extracellular compartments are independent of each other, thus greatly simplifying biophysical modeling.^17,25,26^ However, neglecting transcytolemmal water exchange will lead to a significant underestimation of intracellular volume fractions and cellularity from MR cytometry.^27^ On the other hand, the water exchange rate is highly correlated with the cell membrane permeability, which can reflect pathological changes within tumors.^12,28^ To obtain this important biophysical information, several methods have been proposed recently, such as JOINT^18^ and EXCHANGE.^19^ This prospective study is the first to evaluate the efficacy of JOINT and EXCHANGE, which incorporated transcytolemmal water exchange into biophysical modeling, in predicting IHC factor status and molecular subtypes of breast cancer. We compared these methods with time-dependent ADC measurements and IMPULSED.

## Patients and methods

### Patients

This study was approved by the institution review board of the local hospital. A total of 96 patients with breast cancer who underwent MRI from May 2023 to April 2024 were prospectively collected, with the inclusion criteria as follows: (1) unilateral lesions with a diameter >1 cm; (2) no prior treatment for any breast disease before the MRI examination, including biopsy, neoadjuvant therapy, and other anti-tumor treatments; (3) complete clinical pathological data available after the MRI examination. Six of ninety-six cases were excluded due to failure in fat suppression (n = 3), motion artifacts (n = 2) and multi-focal lesions (n = 1).

### dMRI acquisition

Breast dMRI acquisitions were conducted on a 3T MR scanner with a maximum gradient amplitude of 80 mT/m and a maximum slew rate of 200 T/m/s (MAGNETOM Prisma, Siemens Healthineers, Forchheim, Germany). A dedicated 16-channel phased-array breast coil was employed. A combined acquisition protocol lasting 6 minutes was used to obtain diffusion images with different diffusion times, which included the PGSE (effective diffusion time *t_d_* = 70ms), OGSE 25Hz (*t_d_* = 5ms) and OGSE 50 Hz (*t_d_* = 5ms) sequences. Detailed acquisition information is demonstrated in Supplementary Appendix 1.

### Histopathological examinations

Immunohistochemistry and fluorescence in situ hybridization (FISH) were performed on each participant followed by MRI examination. According to the obtained status (positive or negative) of Estrogen Receptor (ER), Progesterone Receptor (PR), Human Epidermal Growth Factor Receptor-2 (HER2) and proliferation marker (Ki67), the lesions were classified into Luminal A, Luminal B, HER2-enriched and Triple-Negative Breast Cancer (TNBC) subtypes (Supplementary Appendix 2).

### Data analysis

Three MR cytometry methods were used to fit microstructural parameters from the acquired dMRI signals: IMPULSED, JOINT and EXCHANGE, please refer to Supplementary Materials for more details. There are four free parameters in IMPULSED, including intracellular volume fraction *v_in_*, cell diameter *d*, intracellular intrinsic diffusivity *D_in_*, and apparent extracellular diffusivity *D_ex_*. For JOINT and EXCHANGE, water exchange rate constant *k_in_* was introduced as an additional free parameter. *D_in_* was fixed as 1.56 μm^2^/ms to stabilize the fitting procedure, and the number of free parameters remains four: *v_in_, d, D_ex_* and *k_in_*. Cellularity was calculated as. 2(3*v_in_*/2π · 100)^2/3^/*d*^2^.^28^ The data fitting platform, MATI^29^, was used to solve the microstructural parameters. The details of these MR cytometry methods can be found in Supplementary Appendix 3.

For the conventional t_d_-dMRI measurements, ADC values at each sequence were obtained by fitting the multi-b signals to *S* = exp(–*b* · ADC). Besides, ΔADC was calculated as (ADC_50Hz_ – ADC_PGSE_) to quantify the change of ADC with diffusion time.

Regions of interest (ROIs) were manually drawn on the diffusion-weighted images by radiologists L. W. and H. B. (with six years of experience), who were blinded to the final pathological results. Surrounding fatty, muscle, cystic, hemorrhagic and necrotic tissues were carefully excluded from the ROIs. The time-dependent ADC metrics and model-fitted microstructural parameters were calculated within each voxel and then averaged over the whole ROI.

### Statistical analysis

Statistical analysis methods varied by grouping strategies.

#### Grouping by IHC factors

There are four binary classification tasks, *i.e*. ER(+) *vs*. ER(-), PR(+) *vs*. PR(-), HER2(+) *vs*. HER2(-) and Ki67(+) *vs*. Ki67(-). The differences of each metric were evaluated by the rank-sum test (Mann-Whitney U test). The performance in predicting IHC factors were evaluated by logistic regression models in SSPS (Chicago, IL). The area under the receiver operating characteristic (ROC) curve (AUC) was calculated to quantify the predictive efficacy. The classifiers combining different parameters were also evaluated, specifically, (ADC_PGSE_, ADC_25Hz_, ADC_50Hz_, ΔADC) for ADC metrics, (*d, v_in_, D_ex_, D_in_*, cellularity) for IMPULSED, and (*d, v_in_, k_in_, D_ex_*, cellularity) for JOINT and EXCHANGE.

#### Grouping by molecular subtypes

This is a four-class classification task, *i.e*. Luminal A vs Luminal B *vs*. TNBC *vs*. HER2-enriched subtypes. Kruskal-Wallis one-way analysis of variance was used to evaluate the significance of differences in imaging metrics among the four molecular subtypes. Logistic regression analyses was also performed to identify each subtype. Four ROC curves and AUC values were obtained for each metric or their combinations.

## Results

### Patient characteristics

A total of 90 patients with 90 lesions were included in this prospective study. Among these, 26 cases were Luminal A subtype, 38 cases were Luminal B subtype, 8 cases were HER2-enriched subtype, and 18 cases were TNBC subtype. The patient information and lesion characteristics are summarized in Table 1.

**TABLE 1.** Patient information and lesion characteristics

Characteristics	Luminal A (n = 26)	Luminal B (n = 38)	TNBC (n = 18)	HER2-enriched (n = 8)
Age(years)	55.11 ± 8.52	51.16 ± 11.01	52.89 ± 8.45	51.00 ± 9.04
Tumor size(mm)	27.65 ± 6.93	27.37 ± 8.25	27.83 ± 9.93	24.50 ± 6.35
Menstruation state				
Premenopausal women	11	17	5	3
Postmenopausal women	15	21	13	5
Tumor border				
Well-defined	9	10	8	4
ill-defined	17	28	10	4
Tumor sharp				
Oval or round	21	32	11	3
Irregular	5	6	7	5
ER status				
Positive	26	38	0	0
Negative	0	0	18	8
PR status				
Positive	24	29	0	0
Negative	2	9	18	8
HER2 status				
Positive	0	13	0	8
Negative	26	25	18	0
Ki67 status				
Positive	3	27	16	6
Negative	23	11	2	2

1 ER = estrogen receptor; HER2 = human epidermal growth factor receptor 2; Ki67 = nuclear associated antigen; PR = progesterone receptor; TNBC = triple-negative breast cancer

### Microstructural feature mapping of breast tumors

**FIGURE 1.**
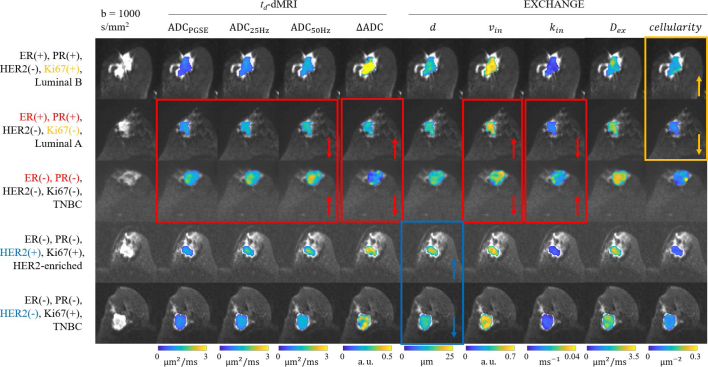
The ADC and microstructural maps overlaid on b = 1000 s/mm^2^ diffusion-weighted images of five representative breast cancer patients. ADC = apparent diffusion coefficient; ER = estrogen receptor; *d* = diameter; *D_ex_* = apparent extracellular diffusivity; *D_in_* = intracellular intrinsic diffusivity; HER2 = human epidermal growth factor receptor 2; Ki67 = nuclear associated antigen; *K_in_* = water exchange rate; TNBC = triple-negative breast cancer; *v_in_* = intracellular volume fraction; ΔADC = (ADC_50Hz_ – ADC_PGSE_) / ADC_PGSE_

Supplementary Figure 1 shows the PGSE and OGSE diffusion-weighted images for a representative patient (Luminal B subtype). The lesion conspicuity is acceptable. Figure 1 shows the ADC metrics and microstructural parameter maps for five representative breast cancer patients with different IHC factor status and molecular subtypes. The overall ADC values decreased with longer diffusion time, *i.e*. ADC_PGSE_ < ADC_25Hz_ < ADC_50Hz_. As shown in the red boxes, the lower ADC and *k_in_*, higher ΔADC and *v_in_* were observed in the ER(+)/PR(+) case compared to the negative case. Besides, *d* was found to be larger in the HER2(+) case. As indicated by the yellow box, higher cellularity was observed in the Ki67(+) case.

### Intergroup comparison

**FIGURE 2.**
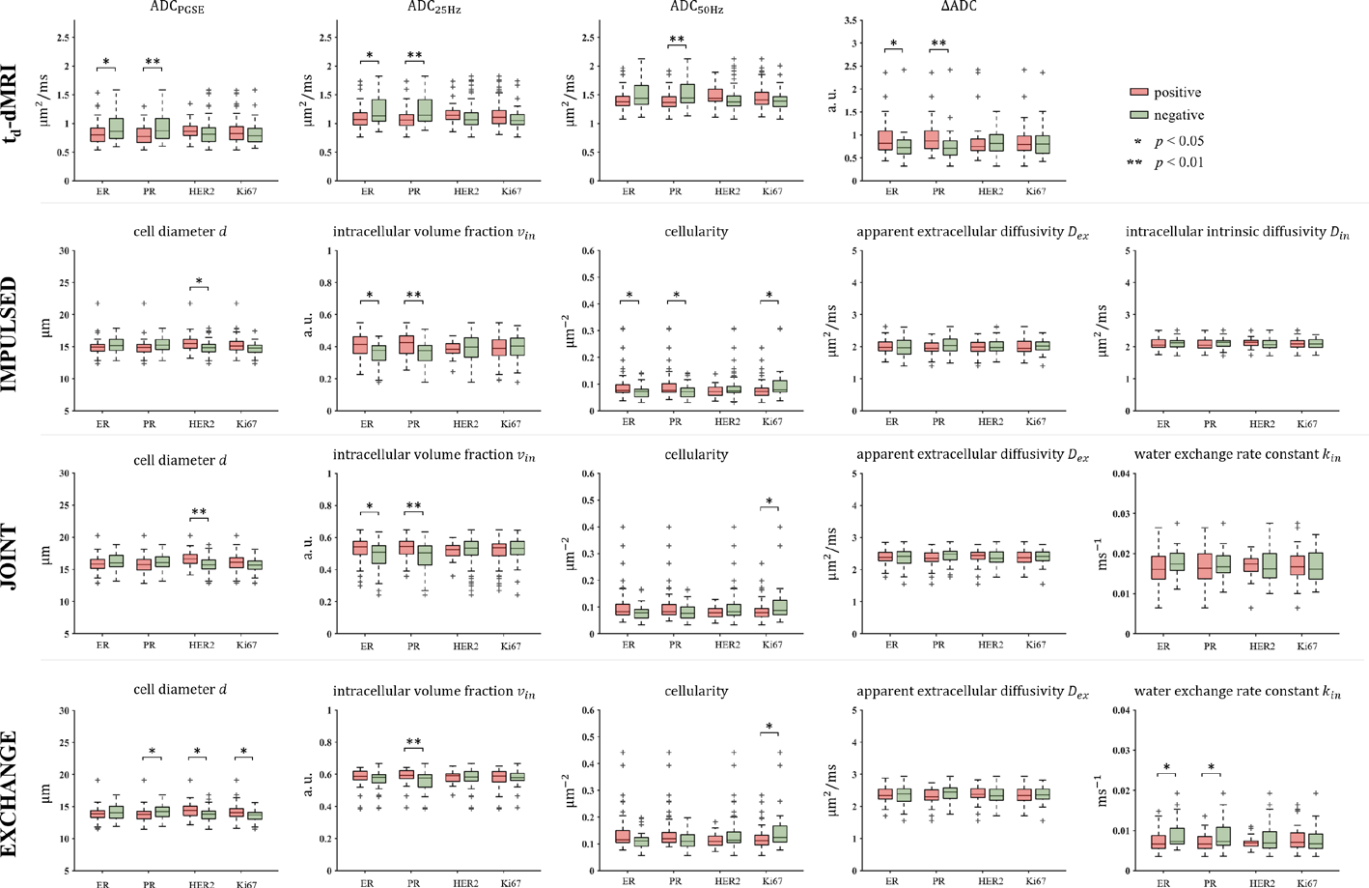
Intergroup comparison of t_d_-MRI metrics and microstructural parameters respectively fitted from IMPULSED, JOINT and EXCHANGE between positive and negative immunohistochemical factor status. * = *p* < 0.05, ** = *p* < 0.01. + represents outliers

Figure 2 shows the results of intergroup comparisons in the IHC factors. For t_d_-dMRI measurements, ADC_PGSE_ and ADC_25Hz_ were significantly lower in ER(+) cases, and was higher (*p* = 0.047, 0.049 and 0.016, respectively). The PR(+) group also showed lower ADC_PGSE_, ADC_25Hz_, ADC_50Hz_ and higher ΔADC (*p* = 0.002, 0.004, 0.005 and 0.002, respectively). In contrast, none ADC-related metrics were significantly different in the comparisons for HER2 and Ki67. For the MR cytometry-derived parameters, increased *v_in_* was shown in the ER(+) group for both IMPULSED and JOINT (*p* = 0.013 and 0.030, respectively), while the EXCHANGE-derived *k_in_* was lower (*p* = 0.012) and the IMPULSED-derived cellularity was higher (*p* = 0.027). For PR, the fitted *v_in_* was also higher in the positive group for all three MR cytometry methods (*p* = 0.003, 0.006 and 0.006, respectively).Additionally, the EXCHANGE-derived and were lower (*p* = 0.047 and 0.022, respectively) and the IMPULSED-derived cellularity was higher (*p* = 0.027) in the PR(+) group. For HER2, the diameters were larger in the positive group, with *p* = 0.035 for IMPULSED, *p* = 0.006 for JOINT, and *p* = 0.038 for EXCHANGE. For Ki67, all three methods obtained lower cellularity (*p* = 0.029, 0.037 and 0.037, respectively) in the positive group, while the EXCHANGE-derived was higher (*p* = 0.026).

Table 2 shows the results of intergroup comparisons across the four molecular subtypes. The cell diameter was the only metric that showed significant difference across the four breast cancer molecular subtypes (*p* = 0.038, 0.031 and 0.025 for IMPULSED, JOINT and EXCHANGE, respectively). This microstructural parameter was found to be the highest in HER2-enriched subtype and lowest in Luminal A subtype.

**TABLE 2.** The intergroup comparison for the imaging metrics across four breast cancer molecular subtypes

Model	Parameter	TNBC Median (IQR)	HER2-enriched Median (IQR)	Luminal A Median (IQR)	Luminal B Median (IQR)	^*p*^
t_d_-dMRI	ADC_PGSE_	0.85 (0.49)	0.90 (0.25)	0.74 (0.25)	0.81 (0.25)	0.106
	ADC_25Hz_	1.10 (0.51)	1.21 (0.31)	1.03 (0.19)	1.08 (0.22)	0.055
	ADC_50Hz_	1.44 (0.52)	1.53 (0.26)	1.38 (0.18)	1.38 (0.18)	0.071
	ΔADC	0.73 (0.38)	0.66 (0.29)	0.81 (0.41)	0.83 (0.42)	0.075
IMPULSE	*d*	15.00 (1.73)	16.16 (1.85)	14.79 (1.33)	14.96 (1.07)	**0.038**
	V_in_	0.38 (0.13)	0.37 (0.07)	0.42 (0.13)	0.41 (0.09)	0.063
	D_ex_	1.91 (0.54)	2.08 (0.28)	2.02 (0.24)	1.95 (0.32)	0.712
	D_in_	2.09 (0.20)	2.15 (0.08)	2.07 (0.31)	2.05 (0.21)	0.598
	Cellularity	0.074 (0.03)	0.058 (0.03)	0.078 (0.05)	0.075 (0.03)	0.071
JOIN	d	15.73 (1.57)	17.17 (2.09)	15.65 (2.02)	16.05 (1.42)	**0.031**
	v_in_	0.51 (0.15)	0.51 (0.09)	0.54 (0.09)	0.55 (0.07)	0.144
	k_in_	18.12 (6.66)	16.38 (2.89)	15.68 (6.75)	16.74 (5.09)	0.374
	D_ex_	2.35 (0.41)	2.54 (0.22)	2.40 (0.26)	2.37 (0.32)	0.596
	Cellularity	0.081 (0.04)	0.063 (0.03)	0.083 (0.06)	0.082 (0.03)	0.114
EXCHANGE	d	13.91 (1.31)	15.07 (1.79)	13.67 (1.20)	13.94 (1.07)	**0.025**
	v_in_	0.58 (0.10)	0.58 (0.07)	0.58 (0.05)	0.59 (0.05)	0.280
	k_in_	8.12 (5.50)	7.00 (3.44)	6.70 (3.9)	6.67 (2.2)	0.061
	D_ex_	2.30 (0.45)	2.52 (0.20)	2.36 (0.31)	2.30 (0.34)	0.442
	Cellularity	0.12 (0.03)	0.09 (0.03)	0.12 (0.08)	0.12 (0.03)	0.053

1 The numbers in bold represent there is a significant difference across four molecular subtypes.

1 ADC = apparent diffusion coefficient; ER = estrogen receptor; *d* = diameter; *D_ex_* = apparent extracellular diffusivity; *D_in_* = intracellular intrinsic diffusivity; HER2 = human epidermal growth factor receptor 2; IQR = Interquartile Range; *k_in_* = water exchange rate; TNBC = triple-negative breast cancer; *v_in_* = intracellular volume fraction; ΔADC = (ADC_50Hz_ – ADC_PGSE_) / ADC_PGSE_

### Predicting immunohistochemistry (IHC) factor status and molecular subtypes

**FIGURE 3.**
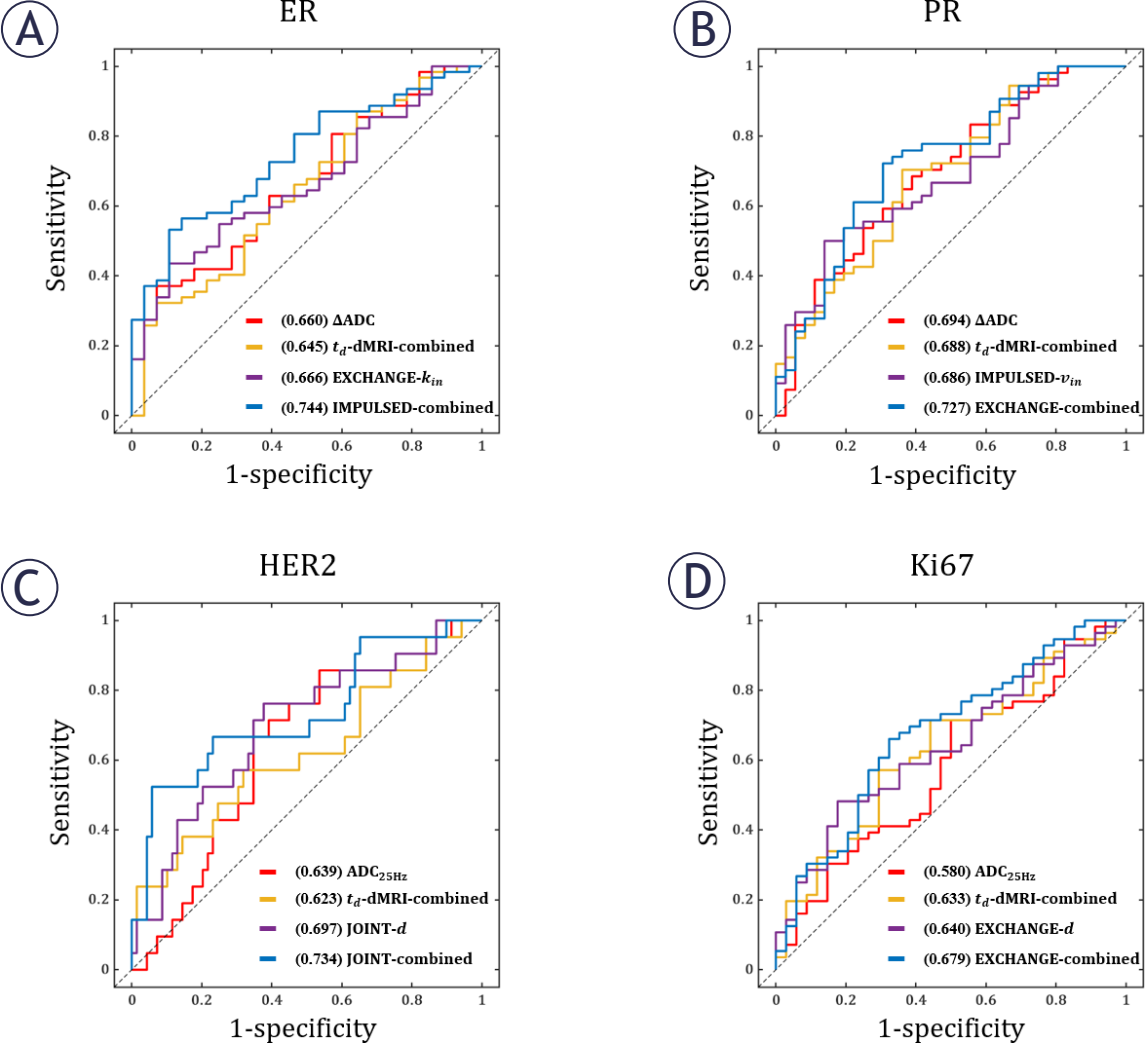
The performance of derived parameters in predicting immunohistochemistry (IHC) factor status. In each sub-plot, the four curves respectively correspond to: the classifier with the highest AUC based on a single td-dMRI metric (ADC_PSGE_, ADC_25Hz_, ADC_50Hz_or ΔADC), the classifier based on the combination of all td-dMRI metrics, the classifier with the highest AUC based on a single model-fitted microstructural parameter (*v_in_, d, k_in_, D_ex_* or *D_in_* obtained from IMPULSED, JOINT, or EXCHANGE), the classifier based on the combination of all parameters obtained from a specific MR cytometry method (IMPULSED, JOINT, or EXCHANGE) that provided the highest combined AUC. **(A)** ER; **(B)** PR; **(C)** HER2; **(D)** Ki67. The numbers within the parentheses in the legend represent the AUC of the corresponding parameters. ADC = apparent diffusion coefficient; ER = estrogen receptor; *d* = diameter; *D_ex_* = apparent extracellular diffusivity; *D_in_* = intracellular intrinsic diffusivity; HER2 = human epidermal growth factor receptor 2; Ki67 = nuclear associated antigen; *k_in_* = water exchange rate; PR = progesterone receptor; *v_in_* = intracellular volume fraction

Table 3 shows the AUC values for the prediction of IHC factor status. For ER, EXCHANGE-derived *k_in_* provided the highest AUC of 0.666 (95% CI, 0.552, 0.781; *p* = 0.012) among the classifiers based on a single imaging metric. By combining multiple microstructural parameters, IMPULSED can improve the AUC to 0.744 (95% CI, 0.641, 0.846; *p* < 0.001). For PR, the highest AUCs based on a single metric and combined regression model were 0.694 (ΔADC, 95% CI, 0.583, 0.806; *p* = 0.002) and 0.727 (EXCHANGE, 95% CI, 0.620, 0.835, *p* < 0.001), respectively. For HER2, the above two highest AUCs were 0.697 (JOINT-derived, 95% CI, 0.567, 0.827, *p* = 0.006) and 0.734 (JOINT, 95% CI, 0.601, 0.867, *p* = 0.001). For Ki67, they were 0.640 (EXCHANGE-derived, 95% CI, 0.525, 0.755, *p* = 0.026) and 0.679 (EXCHANGE,95% CI, 0.565, 0.793, *p* = 0.005). In Figure 3, each sub-plot shows the performance in predicting the status of a specific IHC factor. In each sub-plot, the four curves respectively correspond to: the classifier with the highest AUC based on a single t_d_-dM-RI metric (ADC_PGSE_, ADC_25Hz_, ADC_50Hz_ or ΔADC), the classifier based on the combination of all t_d_-dMRI metrics, the classifier with the highest AUC based on a single model-fitted microstructural parameter (*v_in_, d, k_in_, D_ex_* or *D_in_* obtained from IMPULSED, JOINT, or EXCHANGE), the classifier based on the combination of all parameters obtained from a specific MR cytometry method (IMPULSED, JOINT, or EXCHANGE) that provided the highest combined AUC.

**TABLE 3.** The diagnostic performance of imaging metrics for the prediction of immunohistochemistry (IHC) factor status

Model	Parameter	AUC (ER)	AUC (PR)	AUC (HER2)	AUC (Ki67)
t_d_-dMRI	ADC_PGSE_	0.631 (0.508, 0.755)	0.693 (0.584, 0.803)	0.594 (0.470, 0.718)	0.553 (0.427, 0.678)
	ADC_25Hz_	0.630 (0.508, 0.752)	0.682 (0.571, 0.793)	0.639 (0.055, 0.767)	0.580 (0.458, 0.702)
	ADC_50Hz_	0.624 (0.493, 0.755)	0.674 (0.560, 0.788)	0.627 (0.500, 0.755)	0.571 (0.449, 0.693)
	ΔADC	0.660 (0.540, 0.779)	**0.694 (0.583, 0.806)**	0.468 (0.328, 0.608)	0.496 (0.369, 0.623)
	Combined	0.645 (0.522, 0.768)	0.688 (0.576, 0.800)	0.623 (0.476, 0.770)	0.633 (0.516, 0.750)
IMPULSED	*d*	0.590 (0.454, 0.726)	0.621 (0.501, 0.742)	0.652 (0.512, 0.793)	0.612 (0.494, 0.730)
	V_in_	0.664 (0.550, 0.779)	0.686 (0.576, 0.796)	0.554 (0.433, 0.675)	0.545 (0.419, 0.670)
	D_ex_	0.529 (0.389, 0.669)	0.587 (0.461, 0.714)	0.518 (0.337, 0.659)	0.558 (0.438, 0.679)
	D_in_	0.540 (0.407, 0.673)	0.595 (0.473, 0.716)	0.567 (0.433, 0.700)	0.524 (0.399, 0.649)
	Cellularity	0.646 (0.521, 0.771)	0.638 (0.519, 0.758)	0.567 (0.426, 0.708)	0.638 (0.521, 0.754)
	Combined	**0.744 (0.641, 0.846)**	0.705 (0.597, 0.813)	0.689 (0.552, 0.826)	0.646 (0.532, 0.760)
JOIN	d	0.575 (0.443, 0.707)	0.601 (0.481, 0.721)	**0.697 (0.567, 0.827)**	0.595 (0.476, 0.714)
	v_in_	0.643 (0.523, 0.764)	0.673 (0.559, 0.787)	0.453 (0.330, 0.577)	0.517 (0.394, 0.641)
	k_in_	0.623 (0.507, 0.740)	0.535 (0.415, 0.655)	0.459 (0.335, 0.583)	0.520 (0.392, 0.649)
	D_ex_	0.487 (0.351, 0.623)	0.601 (0.478, 0.724)	0.536 (0.399, 0.673)	0.524 (0.403, 0.646)
	Cellularity	0.619 (0.490, 0.747)	0.613 (0.491, 0.736)	0.577 (0.438, 0.716)	0.632 (0.513, 0.750)
	Combined	0.731 (0.625, 0.837)	0.718 (0.609, 0.827)	**0.734 (0.601, 0.867)**	0.666 (0.552, 0.781)
EXCHANGE	d	0.584 (0.450, 0.718)	0.624 (0.504, 0.744)	0.650 (0.510, 0.790)	**0.640 (0.525, 0.755)**
	v_in_	0.596 (0.466, 0.725)	0.671 (0.555, 0.788)	0.511 (0.380, 0.642)	0.466 (0.343, 0.590)
	k_in_	**0.666 (0.552, 0.781)**	0.643 (0.526, 0.760)	0.528 (0.407, 0.650)	0.547 (0.420, 0.675)
	D_ex_	0.521 (0.382, 0.661)	0.608 (0.483, 0.732)	0.562 (0.424, 0.699)	0.522 (0.401, 0.643)
	Cellularity	0.618 (0.490, 0.745)	0.617 (0.496, 0.739)	0.594 (0.445, 0.732)	0.632 (0.515, 0.748)
	Combined	0.725 (0.610, 0.839)	**0.727 (0.620, 0.835)**	0.668 (0.542, 0.794)	**0.679 (0.565, 0.793)**

1 AUC values are presented as mean (bootstrapped 95% CIs). The numbers in bold represent the highest AUC values respectively achieved by the single-variable regression model and multi-variable (combined) regression model. In the combined model, all the parameters obtained by each method was included.

1 ADC = apparent diffusion coefficient; *d* = diameter; *D_ex_* = apparent extracellular diffusivity; *D_in_* = intracellular intrinsic diffusivity; ER = estrogen receptor; HER2 = human epidermal growth factor receptor 2; Ki67 = nuclear associated antigen; *K_in_* = water exchange rate; PR = progesterone receptor; *V_in_* = intracellular volume fraction; ΔADC = (ADC_50Hz_ – ADC_PGSE_) / ADC_PGSE_

**FIGURE 4.**
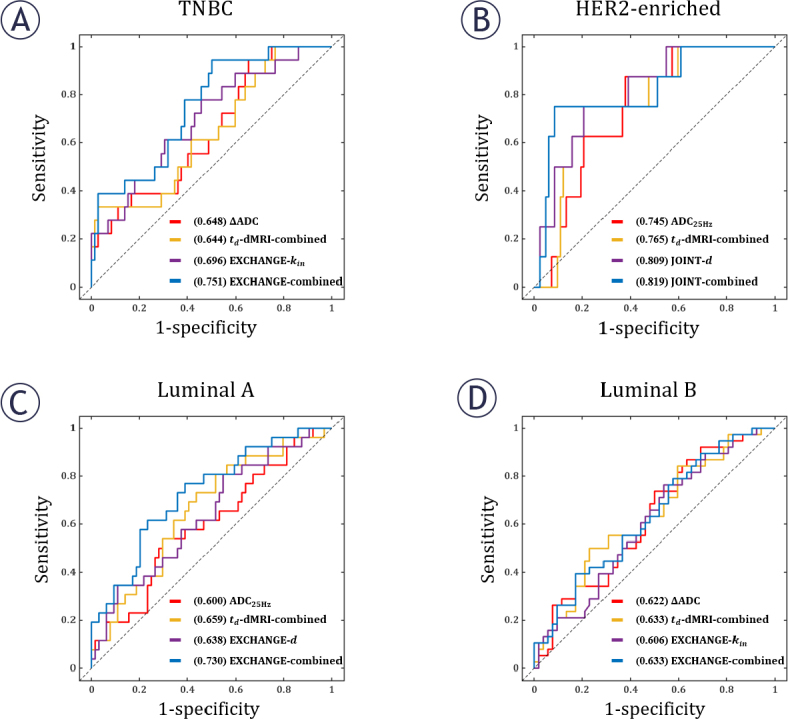
The performance of derived parameters in predicting breast cancer molecular subtypes. In each sub-plot, the four curves respectively correspond to: the classifier with the highest AUC based on a single td-dMRI metric (ADC_PSGE_, ADC_25Hz_, ADC_50Hz_ or ΔADC), the classifier based on the combination of all td-dMRI metrics, the classifier with the highest AUC based on a single model-fitted microstructural parameter (*v_in_, d, k_in_, D_ex_* or *D_in_* obtained from IMPULSED, JOINT, or EXCHANGE), the classifier based on the combination of all parameters obtained from a specific MR cytometry method (IMPULSED, JOINT, or EXCHANGE) that provided the highest combined AUC. (A) TNBC; (B) HER2-enriched; (C) Luminal A; (D) Luminal B. The numbers within the parentheses in the legend represent the AUC of the corresponding parameters. ADC = apparent diffusion coefficient; AUC = area under the receiver operating characteristic curve; TNBC = triple-negative breast cancer; *d* = diameter; *D_ex_* = apparent extracellular diffusivity; *D_in_* = intracellular intrinsic diffusivity; *K_in_* = water exchange rate; PR = progesterone receptor; *V_in_* = intracellular volume fraction

Table 4 shows the AUC values for the prediction of molecular subtypes of breast cancers. For TNBC, EXCHANGE-derived *k_in_* provided the highest AUC of 0.696 (95% CI, 0.561, 0.831, *p* = 0.01) among the classifiers based on a single imaging metric. Combining other microstructural parameters improved the AUC to 0.751 (95% CI, 0.633, 0.869, *p* = 0.001). For HER2-enriched, the highest AUCs based on a single metric and combined model were 0.809 (JOINT-derived *d*, 95% CI, 0.675, 0.944, *p* = 0.004) and 0.819 (JOINT, 95% CI, 0.657, 0.980, *p* = 0.003). For Luminal A, the above two values were 0.638 (EXCHANGE-derived, 95% CI, 0.513, 0.764, *p* = 0.041) and 0.730 (EXCHANGE, 95% CI, 0.616, 0.843, *p* = 0.001). For Luminal B, they are 0.622 (ΔADC, 95%CI, 0.506, 0.738, *p* = 0.049) and 0.633 (EXCHANGE, 95% CI, 0.518, 0.748, *p* = 0.032). In Figure 4, each subplot shows the performance in predicting a specific molecular subtype. The representative ROC curves are selecteD_in_ the same way as in Figure 3.

**TABLE 4.** The diagnostic performance of imaging metrics for the prediction of molecular subtypes

Model	Parameter	AUC (TNBC)	AUC (HER2- enriched)	AUC (Luminal A)	AUC (Luminal B)
ADC	ADC_PGSE_	0.617 (0.470, 0.763)	0.681 (0.519, 0.844)	0.570 (0.438, 0.703)	0.577 (0.458, 0.697)
	ADC_25Hz_	0.518 (0.435, 0.727)	0.745 (0.614, 0.877)	0.600 (0.470, 0.729)	0.551 (0.429, 0.672)
	ADC^50Hz^	0.575 (0.411, 0.739)	0.744 (0.624, 0.863)	0.576 (0.449, 0.703)	0.566 (0.446, 0.686)
	ΔADC	0.648 (0.511, 0.785)	0.360 (0.141, 0.579)	0.474 (0.340, 0.609)	**0.622 (0.506, 0.738)**
	Combined	0.644 (0.501, 0.786)	0.765 (0.623, 0.907)	0.659 (0.538, 0.781)	0.633 (0.517, 0.748)
IMPULSED	*d*	0.519 (0.316, 0.676)	0.784 (0.609, 0.958)	0.614 (0.487, 0.741)	0.490 (0.370, 0.610)
	V_in_	0.657 (0.522, 0.793)	0.651 (0.489, 0.813)	0.572 (0.433, 0.711)	0.593 (0.475, 0.710)
	D_ex_	0.537 (0.367, 0.707)	0.582 (0.375, 0.790)	0.565 (0.445, 0.684)	0.558 (0.436, 0.680)
	D_in_	0.507 (0.348, 0.666)	0.622 (0.468, 0.776)	0.514 (0.376, 0.653)	0.533 (0.412, 0.654)
	Cellularity	0.593 (0.447, 0.738)	0.720 (0.503, 0.936)	0.606 (0.474, 0.737)	0.455 (0.336, 0.574)
	Combined	0.748 (0.629, 0.868)	0.739 (0.531, 0.947)	0.666 (0.544, 0.789)	0.630 (0.513, 0.747)
JOIN	d	0.519 (0.367, 0.671)	**0.809 (0.675, 0.944)**	0.590 (0.460, 0.719)	0.515 (0.394, 0.635)
	v_in_	0.644 (0.496, 0.791)	0.611 (0.438, 0.785)	0.545 (0.412, 0.678)	0.593 (0.475, 0.712)
	k_in_	0.630 (0.489, 0.772)	0.486 (0.349, 0.624)	0.558 (0.414, 0.701)	0.541 (0.420, 0.663)
	D_ex_	0.521 (0.363, 0.679)	0.642 (0.438, 0.845)	0.507 (0.383, 0.631)	0.539 (0.417, 0.662)
	Cellularity	0.549 (0.396, 0.703)	0.733 (0.537, 0.929)	0.584 (0.450, 0.718)	0.461 (0.342, 0.580)
	Combined	0.742 (0.616, 0.869)	**0.819 (0.657, 0.980)**	0.648 (0.525, 0.770)	0.609 (0.492, 0.727)
EXCHANGE	d	0.509 (0.357, 0.661)	0.784 (0.602, 0.965)	**0.638 (0.513, 0.764)**	0.516 (0.396, 0.636)
	v_in_	0.627 (0.477, 0.778)	0.532 (0.309, 0.755)	0.492 (0.364, 0.621)	0.601 (0.481, 0.721)
	k_in_	**0.696 (0.561, 0.831)**	0.459 (0.299, 0.618)	0.543 (0.402, 0.684)	0.606 (0.489, 0.723)
	D_ex_	0.478 (0.313,0.644)	0.666 (0.468, 0.865)	0.514 (0.390, 0.637)	0.553 (0.431, 0.674)
	Cellularity	0.542 (0.393, 0.692)	0.756 (0.559, 0.953)	0.620 (0.490, 0.750)	0.488 (0.368, 0.608)
	Combined	**0.751 (0.633, 0.869)**	0.784 (0.598, 0.969)	**0.730 (0.616, 0.843)**	**0.633 (0.518, 0.748)**

1 AUC values are presented as mean (bootstrapped 95% CIs). The numbers in bold represent the highest AUC values respectively achieved by the single-variable regression model and multi-variable (combined) regression model. In the combined model, all the parameters obtained by each method was included.

1 ADC = apparent diffusion coefficient; *d* = diameter; *D_ex_* = apparent extracellular diffusivity; *D_in_* = intracellular intrinsic diffusivity; *K_in_* = water exchange rate; PR = progesterone receptor; TNBC = triple-negative breast cancer; *V_in_* = intracellular volume fraction; ΔADC = (ADC_50Hz_ – ADC_PGSE_) / ADC_PGSE_

## Discussion

Determining the IHC factor status and molecular subtypes of breast cancer is an important reference for the development of appropriate clinical treatment regimes. The dMRI-derived ADC metrics have shown potential in the prediction of the IHC factor status and molecular subtypes^10^, without the injection of contrast agents in DCE MRI. However, the results of previous studies are controversial. Several publications^10,30,31^ reported that the ADC values were lower in ER(+) and PR(+) breast cancers, whereas Park *et al*.^32^ showed no significant differences in ADC values between ER(+) and ER(-), as well as PR(+) and PR(-). Besides, some studies^30–33^ demonstrated the higher ADC in the HER2(+) group, whereas others have shown the opposite results^34^ or no significant difference^.35^ For Ki67, the conclusion also remains uncertain. Shen *et al*.^36^ found that the ADC metrics decreased with higher Ki-67 labeling index, while other findings^33,37^ showed that there was no significant difference between high and low Ki67 expression. Some advanced methods, such as intra-voxel incoherent motion (IVIM) imaging^38,39^ and diffusionkurtosis imaging (DKI)^39^, can improve the efficacy to distinguish the status of ER and PR, but failed to differentiate HER2 and Ki67. Recently, Lima *et al*.^40^ utilized time-dependent ADC measurements obtained by PGSE and OGSE to characterize breast cancer based on IHC markers. Furthermore, Ba *et al*.^21^ and Wang *et al*.^22^ have implemented the emerging MR cytometry method IMPULSED to extract quantitative microstructural information of breast tumors, and the derived parameters has been shown to be effective in the prediction of IHC factor status, molecular subtypes and treatment response to neoadjuvant chemotherapy. However, the biophyscial model used in IMPULSED neglected transcytolemmal water exchange, resulting in the underestimation of *v_in_* and unavailability of membrane permeability.^27^ This study investigated the efficacy of the MR cytometry methods that incorporate water exchange in predicting IHC factor status and molecular subtypes. The systematic comparisons between the different MR cytometry methods and the t_d_-dMRI measurements provide guidance for clinical application of MR cytometry.

In this study, the lower ADC_PGSE_ and ADC_25Hz_ values and higher ΔADC were observed in the ER(+) group compared to ER(-), similarly, all three ADC metrics were lower and ΔADC values were higher in the PR(+) group, which are consistent with the previous results.^30,31,40^ Some studies^40,41^ suggested that the lower ADC values for ER(+) and PR(+) may be due to lower cell membrane permeability, which has been reflected in the results of microstructural parameters, the EXCHANGE-derived transcytolemmal water exchange rate constant *k_in_* was lower in the ER(+) and PR(+) groups, indicating lower membrane permeability. In addition, the IMPULSED and JOINT-derived *v_in_* was larger in the ER(+) group, and all three quantitative methods-derived *v_in_* was larger in the PR(+) group, which may be related to the increased pathological cellularity with ER or PR overexpression in breast tumors.^42^ For the intergroup comparison between HER2(+) and HER2(-), there was no significant difference in ADC-related metrics, this may be due to the fact that HER2 overexpression leads to both increased cell proliferation and angiogenesis, whereas they have opposite impacts on ADC values.^30^ However, the cell diameter *d* obtained from all three MR cytometry methods was significantly larger in the HER2(+) group, which was consistent with the pathological finding that HER2-overexpressing breast cancer has increased cell size.^43^ Finally, for the prediction of Ki67 factor, only cellularity and the EXCHANGE-derived showed significant difference, but we are still unclear about the reasons behind this. On the other hand, for different molecular subtypes, the model-fitted values were the only metrics exhibiting significant difference among these subtypes. Larger *d* was observed in HER2-enriched subtype compared to non-HER2-enriched subtype, which may be attributed to the HER2 overexpression (larger in the HER(+) group).

In this study, we also systemically compared the diagnosis performance of three MR cytometry methods in predicting IHC factor status and molecular subtypes. For the classifiers based on a single metric, provided the highest AUC in the prediction of PR status and Luminal B subtype; JOINT obtained the highest AUC in predicting HER2 status and HER2-enriched subtype; EXCHANGE performed best in predicting ER, Ki67 status, TNBC and Luminal A subtypes. For the classifiers based on the combined regression model, IMPULSED provided the highest AUC in predicting ER status; JOINT obtained the highest AUC in the prediction of HER2 status and HER2-enriched subtype; EXCHANGE achieved the highest AUC in the prediction of PR status, Ki67 status, TNBC, Luminal A and Luminal B subtypes. The above results show that MR cytometry methods may provide better diagnostic efficacy in the prediction of IHC factor status and molecular subtypes, compared to traditional t_d_-dMRI measurements. Meanwhile, the MR cytometry methods incorporating water exchange (JOINT and EXCHANGE) improved the diagnostic efficacy compared to IMPULSED (except for ER status). Although previous numerical simulation and in vitro cell experiments^18,19^ demonstrated that JOINT and EXCHANGE, which incorporated water exchange, obtained more accurate estimation of *v_in_* and an additional biophysical parameter *k_in_*, our study found only minor improvements in breast cancer subtyping when water exchange is incorporate into MR cytometry. Thus, while it is desirable to incorporate such objective biophysical phenomena into the biophysical model, improved model accuracy does not necessarily translate into superior clinical diagnostic performance.

There are several limitations in this study. First, the data were collecte in a single center with limited sample size, especially the HER2-enriched subtype. It is necessary to include more breast cancer patients from multiple hospitals or institutions and validate the results more comprehensively. Second, our study lacks the correlation analysis between the MR cytometry-derived parameters and histopathological results. Such analysis will provide more reliable validation on the imaging results and more comprehensive comparisons between the quantitative methods which will be includeD_in_ our future work. Third, the b values of the OGSE sequence with 50Hz were relatively low (≤500s/mm^2^) due to the limitations of gradient performance. Despite our best efforts to eliminate the impact of IVIM before model fitting, molecular markers of angiogenesis such as micro-vessel density^44^ still introduce bias in the estimation of microstructural parameters, especially when using low b values. Fortunately, the modern whole-body ultra-high-performance gradients can provide higher b values for high-frequency OGSE when PNS allows. Fourth, each imaging metric was averaged across the whole ROI, which lost the information of spatial heterogeneity within breast tumors. Surviving cells, dead cells and necrotic regions may co-exist in each ROI. This spatial heterogeneity can be captured by histogram analysis.^38^

In summary, this study was the first to evaluate the clinical performance of MR cytometry incorporating water exchange in predicting IHC factor status and molecular subtypes of breast cancer, and comprehensively compared the derived microstructural parameters obtained and conventional t_d_-dMRI metrics. Our results showed that advanced MR cytometry outperformed traditional ADC measurements. Incorporating water exchange into MR cytometry methods further improved the diagnosis performance. Specifically, the results based on the multi-variable regression models showed that: IMPULSED performed best in predicting ER status; JOINT was more suitable for predicting HER2 status and HER2-enriched subtype; EXCHANGE can provide the highest AUC in predicting PR and Ki67 status, TNBC, Luminal A and Luminal B subtypes.

## Supplementary Material

Supplementary Material Details
