# Supplementary material for: Comparison of MR cytometry methods in predicting immunohistochemical factor status and molecular subtypes of breast cancer
Source: Radiol Oncol. 2025 Aug 6;59(3):337–48. doi: 10.2478/raon-2025-0044 (PMC12441884; doi:10.2478/raon-2025-0044)
Supplement: Supplementary file 1 — Supplementary Material Details [file raon-2025-0044_sm.pdf]

Comparison of MR cytometry methods in predicting immunohistochemical factor status and molecular subtypes of breast cancer

Lei Wu, Fan Liu, Sisi Li, Xinyi Luo, Yishi Wang, Wen Zhong, Thorsten Feiweier, Junzhong Xu, Haihua Bao, Diwei Shi, Hua Guo

doi: 10.2478/raon-2025-0044

Supplementary Appendix 1

MRI acquisition

A combined acquisition protocol lasting 6 minutes was used to obtain diffusion images with different diffusion times, which included the PGSE and OGSE sequences. A PGSE sequence with diffusion gradient duration δ / diffusion gradient separation Δ = 12/74 ms was used to acquire images at relatively long diffusion time (70ms). OGSE sequences at 25Hz and 50Hz were used to acquire images at shorter diffusion times (10 and 5 ms). For PGSE and OGSE at 25Hz, five b values (0, 250, 500, 750, 1000 s/mm^2) were used. Three b values (0, 250, 500 s/mm^2) were used for OGSE at 50Hz. Other acquisition settings include: a 2D single-shot echo-planar imaging (EPI); three diffusion directions; FOV = 340×192 mm^2; in-plane resolution = 2.0×2.0 mm^2; slice thickness = 4.0 mm; 10 slices; GRAPPA factor = 2; partial Fourier factor = 7/8; echo spacing = 0.53 ms; bandwidth = 2262 Hz/pixel; TE/TR = 117 / 5000 ms.

Clinical routine sequences were also acquired, including a axial T1-weighted imaging without fat suppression, coronal non-fat-saturated T1-weighted imaging, axial fat-saturated T2-weighted imaging, high-resolution diffusion-weighted imaging RESOLVE (voxel size = 1.6×1.6×5 mm^3), and T1-weighted dynamic contrast-enhanced (DCE) imaging. Notably, all dMRI acquisitions were performed prior to the DCE MRI sequence.

Supplementary Appendix 2

Histopathological examination

According to the IHC results, we obtained the expression of ER, PR, HER2, and Ki67 related to tumor cell proliferation. For ER and PR status, nuclear staining of less than 1% indicates a negative result for both ER and PR. Conversely, nuclear staining exceeding 1% signifies a positive result. For HER2 status, IHC results of negative or 1+ are classified as HER2-negative, while 3+ is considered HER2-positive. In cases with a HER2 expression of 2+, further analysis using fluorescence in situ hybridization (FISH) is conducted. Those exhibiting gene amplification are deemed HER2-positive, while those without are classified as HER2-negative. For Ki67, nuclear staining of 20% or less is considered negative, whereas more than 20% is considered positive.

Typically, breast cancer was categorized into four subtypes based on the IHC and FISH results. The specific criteria for each subtype are as follows:

- Luminal A: ER and/or PR positive, HER2 negative, and Ki67 negative.
- Luminal B: ER and/or PR positive, HER2 positive; ER and/or PR positive, HER2 negative, and Ki67 positive.
- HER2-enriched: ER and PR negative, HER2 positive, and Ki67 positive/negative.
- TNBC: ER, PR, and HER2 all negative, and Ki67 positive/negative.

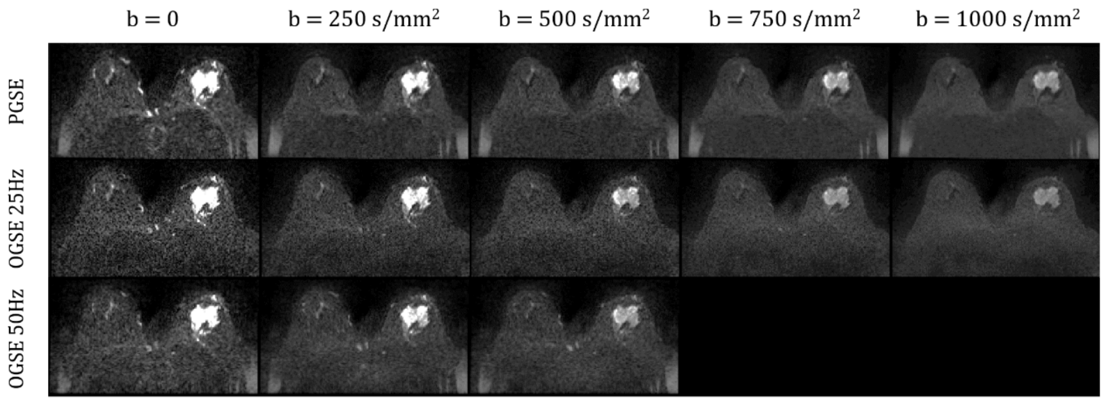

SUPPLEMENTARY FIGURE 1. Representative images acquired by PGSE and OGSE at 25Hz/50Hz.

Supplementary Appendix 3

Analytical expression of intracellular apparent restricted diffusion coefficient

Diffusion-weighted signal assuming a Gaussian phase distribution can be written as:

$$S = S_0 \exp(-\phi) \qquad [S1]$$

where S_0 is the non-diffusion-weighted signal and the echo attenuation factor ϕ can be expressed as the following form based on the velocity correlation function developed by Stepisnik:

$$\phi = \frac{\gamma^2}{2} \sum_k B_k \int_0^{\mathrm{TE}} dt_1 \int_0^{\mathrm{TE}} dt_2 \exp(-a_k D|t_1 - t_2|)\, g(t_1) g(t_2) \qquad [S2]$$

where γ is the gyromagnetic ratio, TE is the echo time, $g(t)$ is the time-varying diffusion gradient, D is the intrinsic diffusion coefficient, B_k and a_k are microstructure-related coefficients, which have been obtained for special shaped boundary conditions such as cylinders and spheres. Based on the Eq.[S2], the analytical expression of restricted dMRI signals under diffusion sequences with arbitrary gradient waveform can be derived, both for the sine and cosine-modulated OGSE and traditional PGSE sequences.

For the PGSE with trapezoid-shaped gradient waveforms, the analytical expression of ϕ is given as:

$$\phi = \gamma^2 G^2 \sum_k \frac{B_k}{a_k^4 D^4 t_r^2} \left\{ \begin{array}{c} 2\exp(-a_k D t_p) - 4\exp(-a_k D t_r) - 4\exp(-a_k D \Delta) \\ +2\exp(-a_k D(\Delta - t_r)) + 2\exp(-a_k D(\Delta + t_r)) \\ -\exp\left(-a_k D(\Delta - t_p)\right) - \exp\left(-a_k D(\Delta + t_p)\right) \\ -4\exp\left(-a_k D(t_r + t_p)\right) + 2\exp\left(-a_k D(2t_r + t_p)\right) \\ +2\exp\left(-a_k D(\Delta - t_r - t_p)\right) + 2\exp\left(-a_k D(\Delta + t_r + t_p)\right) \\ -\exp\left(-a_k D(\Delta - 2t_r - t_p)\right) - \exp\left(-a_k D(\Delta + 2t_r + t_p)\right) \\ -4a_k D t_r + \frac{4}{3} a_k^3 D^3 t_r^3 + 2a_k^3 D^3 t_r^2 t_p + 4 \end{array} \right\} \quad \text{[S3]}$$

where t_r is the gradient rise time and t_p is the duration of each gradient plateau. Similarly, the analytical expression for the cosine-modulated trapezoidal OGSE sequences can also be derived from the Eq. [S3], these results have been shown previously.

In this study, tumor cells were modeled as ideal spheres, then the coefficients B_k and a_k can be expressed as:

$$B_k = \frac{2(R/\mu_k)^2}{\mu_k - 2}, \qquad a_k = \left(\frac{\mu_k}{R}\right)^2 \quad \text{[S4]}$$

where μ_k is the kth root of $\mu J'_{3/2}(\mu) - \frac{1}{2} J_{3/2}(\mu) = 0$ and R is the tumor cell radius. For the intracellular diffusion, the restricted dMRI signal S_r can be expressed as:

$$S_r = S_0 \exp(-\phi) = S_0 \exp(-b \cdot \mathrm{ADC_r}) \quad \text{[S5]}$$

where b is the diffusion-weighted factor (b value) and $\mathrm{ADC_r}$ is the apparent restricted diffusion coefficient, then it can be calculated as:

$$\mathrm{ADC_r} = \frac{\phi}{b} \quad \text{[S6]}$$

Based on Eq. [S6] and [S3], we can obtain the analytical expression of $\mathrm{ADC_r}$, which is related to the cell diameter d or radius R, intracellular intrinsic diffusivity D_{in} ($D = D_{in}$), and specific diffusion-weighted sequences (PGSE and OGSE in this study).

IMPULSED

In this MR cytometry method, the transcytolemmal water exchange between intra- and extracellular compartments has been neglected, where the tumor cells are modeled as impermeable spheres. Then the dMRI signals are modeled as the sum of signals arising from the intra- and extracellular compartments:

$$S = S_{in} + S_{ex} \quad \text{[S7]}$$

where the water diffusion is restricted and hindered in the intra- and extracellular compartments, respectively, the corresponding signal attenuation is:

$$S_{in} = S_{in,0} \exp(-b \cdot \mathrm{ADC_r}) = v_{in} S_0 \exp(-b \cdot \mathrm{ADC_r}) \quad \text{[S8]}$$

and

$$S_{ex} = S_{ex,0} \exp(-b \cdot D_{\mathrm{ex}}) = v_{ex} S_0 \exp(-b \cdot D_{\mathrm{ex}}) \quad \text{[S9]}$$

where $S_{in,0}$ and $S_{ex,0}$ are non-diffusion-weighted signals, v_{in} and v_{ex} are the volume fractions of intra- and extracellular compartments, and D_{ex} is the extracellular hindered diffusivity. Note that $v_{in} + v_{ex} = 1$, the overall signal S can be expressed as:

$$\mathrm{S} = \mathrm{S_0}(\mathrm{v_{in}} \cdot \exp(-\mathrm{b} \cdot \mathrm{ADC_r}) + (1 - \mathrm{v_{in}}) \cdot \exp(-\mathrm{b} \cdot \mathrm{D_{ex}})) \quad \text{[S10]}$$

JOINT

This method has incorporated transcytolemmal water exchange into the IMPULSED analytical framework. The magnetization exchange between the two compartments (S_{in} and S_{ex}) caused by water exchange can be quantified by the modified Kärger model:

$$\begin{aligned} \frac{dS_{in}}{dt} &= -\gamma^2 g^2 \delta^2 \cdot \mathrm{ADC_r} \cdot S_{in} - k_{in}S_{in} + k_{ex}S_{ex} \\ \frac{dS_{in}}{dt} &= -\gamma^2 g^2 \delta^2 \cdot D_{ex} \cdot S_{in} - k_{ex}S_{ex} + k_{in}S_{in} \end{aligned} \qquad [S11]$$

where g and δ are the strength and duration of the diffusion gradient for the PGSE sequences, k_{in} and k_{ex} are the exchange rate constants of magnetizations (from 'in' to 'ex' and from 'ex' to 'in'). By solving the differential Eq. [S11], the diffusion-weighted signal S can be expressed as the following linear combination of exponential terms:

$$S = S_{in} + S_{ex} = S_0(V_1 \exp(-bD_1^*) + (1 - V_1)\exp(-bD_2^*)) \qquad [S12]$$

where the terms D_1^*, D_2^*, and V_1 are:

$$\begin{aligned} D_1^* &= \frac{A_{in} - A_{ex} - D_Q}{2} \\ D_2^* &= \frac{A_{in} - A_{ex} + D_Q}{2} \\ V_1 &= 1 - \frac{\left(A_{ex} - A_{in} + D_Q - 2k_{ex}/\gamma^2 g^2 \delta^2\right)v_{ex} + \left(A_{in} - A_{ex} + D_Q - 2k_{in}/\gamma^2 g^2 \delta^2\right)v_{in}}{2D_Q} \end{aligned} \qquad [S13]$$

The other unknown parameters are computed by:

$$\begin{aligned} A_{in} &= \mathrm{ADC_r} + \frac{k_{in}}{\gamma^2 g^2 \delta^2} \\ A_{ex} &= D_{ex} + \frac{k_{ex}}{\gamma^2 g^2 \delta^2} \\ D_Q &= \sqrt{(A_{in} - A_{ex})^2 + \frac{4k_{in}k_{ex}}{\gamma^4 g^4 \delta^4}} \end{aligned} \qquad [S14]$$

Substituting Eqs. [S14] and [S13] into [S12], we can obtain the final analytical expression after introducing the effect of transcytolemmal water exchange. Note that in the JOINT method, only the PGSE signals are expressed using Eq. [S12], while the other OGSE signals are still represented by Eq. [S10], since this MR cytometry method assumes that water exchange has a limited impact on signal acquisitions for the OGSE sequences with short diffusion times.

EXCHANGE

JOINT makes a strong approximation, i.e., transcytolemmal water exchange influences PGSE acquisitions only, not on OGSE. Due to this reason, numerical simulations and in vitro cell experiments have shown that the accuracy of the JOINT-derived parameters was only valid with relatively slow water exchange. The EXCHANGE method is also based on the Kärger model and IMPULSED analytical framework, but removes the approximation used in JOINT.

Specifically, first, a two-mode diffusion model is proposed to describe the actual intracellular diffusion, which includes not only restricted but also hindered diffusion in the presence of transcytolemmal water exchange. In this model, the probability of an intracellular molecule crossing

the membrane and then moving to the extracellular space is defined as p, and it can be estimated from the existing parameters, including R, D_{in}, and k_{in}:

$$p = \frac{\left(\frac{4R}{3}\right)^2}{\left(\frac{4R}{3}\right)^2 + \frac{2D_{in}}{k_{in}} - \left(\frac{3R}{4}\right)^2} \qquad [S15]$$

Then, the diffusion of intracellular water molecules is divided into two modes: for molecules that stay inside the cell, i.e., restricted diffusion, $\mathrm{ADC_r}$ is used to describe the intensity of the diffusion movement; For molecules that cross the membrane, leave the cell, and undergo hindered diffusion, an average hindered diffusivity D_{inh} is introduced and approximated as a linear combination of $\mathrm{ADC_r}$ and D_{ex}, with the volume fractions v_{in} and v_{ex} as the weights, i.e., $D_{inh} = v_{in}\mathrm{ADC_r} + v_{ex}D_{ex}$. The two-mode diffusion coefficient of the intracellular compartment D_{in}^* can be calculated by the following approximation:

$$D_{in}^* = -\frac{\ln\left((1-p)\exp(-b \cdot \mathrm{ADC_r}) + p\exp(-b \cdot D_{inh})\right)}{b} \qquad [S16]$$

Finally, replace the $\mathrm{ADC_r}$ in Eq. [S11] with D_{in}^*.

Second, a dimensional-analysis-based expression is used to correct the restriction-induced edge-enhancement effect. As shown in our previous work, the actual exchange rate constants of magnetizations k_{in}^m and k_{ex}^m are usually unequal to those of water molecules (k_{in} and k_{ex}), and typically $k_{in}^m > k_{in}$ (which is termed "edge-enhancement effect"). A modified form of k_{in}^m based on the dimensional analysis has been constructed:

$$k_{in}^m = k_{in} \cdot \left(1 + \alpha \cdot (k_{in}bd^2)^{\gamma_1} \cdot \left(\frac{k_{in}d^2}{D_{in}}\right)^{\gamma_2} \cdot (v_{in})^{\gamma_3}\right) \qquad [S17]$$

where the constants $(\alpha, \gamma_1, \gamma_2, \gamma_3)$ are equal to (2.39, 0, 0.83, 2.88), (2.35,0.045,0.58, 3), and (1.7, 0.12, 0.48, 3) for the used PGSE, OGSE N=1 and N=2 sequences, respectively. On the other hand, the extracellular space is regarded as a narrow interstitial space, and there is an approximation that: $k_{ex}^m \approx k_{ex} = k_{in}v_{in}/v_{ex}$.

Third, a discretization-based computational framework is proposed to obtain the restricted dMRI signals and the corresponding $\mathrm{ADC_r}$ under arbitrary gradient waveforms, thus improving the adaptability of EXCHANGE. Based on the Eq. [S2] and discretization of the gradient waveform (Supplementary Figure 2), the exponential attenuation factor ϕ can be expressed as:

$$\phi = \frac{\gamma^2}{2}\sum_k B_k \sum_{i=1}^{M}\sum_{j=1}^{M}\int_{t_{i-1}}^{t_i} \mathrm{d}t_1 \int_{t_{j-1}}^{t_j} \mathrm{d}t_2 \exp(-a_k D_{in}|t_2 - t_1|)\, g(t_1)g(t_2) \qquad [S18]$$

where $t_i = i\tau$. Then a series of $M \times M$ symmetric matrices $\mathbf{C}^k$ are defined, whose elements are:

$$C_{ij}^k = \int_{t_{i-1}}^{t_i} \mathrm{d}t_1 \int_{t_{j-1}}^{t_j} \mathrm{d}t_2 \exp(-a_k D_{in}|t_2 - t_1|)\, g(t_1)g(t_2) \qquad [S19]$$

Each element C_{ij}^k can be calculated by the following approach: first, approximate $g(t)$ during each short pulse as:

$$g(t) \approx g\left(t_{i-1} + \frac{\tau}{2}\right), \text{for: } t_{i-1} < t < t_i \text{ (i.e. } t_{i-1} + \tau) \qquad [S20]$$

Then for $i < j$:

$$\begin{aligned} C_{ij}^k = C_{ji}^k &= \int_{t_{i-1}}^{t_i} g(t_1)\exp(a_k D_{in} t_1)\,\mathrm{d}t_1 \cdot \int_{t_{j-1}}^{t_j} g(t_2)\exp(-a_k D_{in} t_2)\,\mathrm{d}t_1 \\ &= \frac{2(\cosh(a_k D_{in}\tau) - 1)}{(a_k D_{in})^2} g\left(t_{i-1} + \frac{\tau}{2}\right) g\left(t_{j-1} + \frac{\tau}{2}\right) \exp\left(-a_k D_{in}(t_j - t_i)\right) \end{aligned} \qquad [S21]$$

And for $i = j$:

$$C_{ii}^k = \int_{t_{i-1}}^{t_i} dt_1 \int_{t_{i-1}}^{t_i} dt_2 \exp(-a_k D_{in}|t_2 - t_1|)\, g(t_1) g(t_2)$$
$$= \frac{2g\left(t_{i-1} + \frac{\tau}{2}\right)^2}{(a_k D_{in})^2} (a_k D_{in}\tau + \exp(-a_k D_{in}\tau) - 1) \qquad [S22]$$

Based on Eqs. [S21] and [S22], the attenuation factor ϕ can be easily computed as:

$$\phi = \frac{\gamma^2}{2} \sum_k B_k \sum_{i=1}^{M} \sum_{j=1}^{M} C_{ij}^k = \frac{\gamma^2}{2} \sum_k B_k \mathrm{sum}(\mathbf{C}^k) \qquad [S23]$$

where "sum" means to sum all matrix elements. In addition, the b-value can be expressed as:

$$b = \gamma^2 \int_0^{\mathrm{TE}} \left(\int_0^t g(t')\mathrm{d}t' \right)^2 \mathrm{d}t \qquad [S24]$$

Then introduce an auxiliary function $f(t)$:

$$f(t) = \int_0^t g(t')\mathrm{d}t' \qquad [S25]$$

Then the b-value can be rewritten as:

$$b = \gamma^2 \int_0^{\mathrm{TE}} f(t)^2 \mathrm{d}t = \gamma^2 \sum_{i=1}^{M} \int_{t_{i-1}}^{t_i} f(t)^2 \mathrm{d}t \approx \gamma^2 \sum_{i=1}^{M} \frac{\tau}{2} (f(t_{i-1})^2 + f(t_i)^2) \qquad [S26]$$

where:

$$f(t_i) = f(i\tau) = \sum_{j=1}^{i} \int_{t_{j-1}}^{t_j} g(t')\mathrm{d}t' \approx \sum_{j=1}^{i} \tau g\left(t_{i-1} + \frac{\tau}{2}\right) \qquad [S27]$$

Based on Eq. [S27], the b-value can be computed by Eq. [S26] for arbitrary gradient waveforms. Then the apparent restricted diffusion coefficient $\mathrm{ADC_r}$ can be calculated using Eq. [S6].

Simulation in silico, cells in vitro and animal studies have shown that EXCHANGE can provide accurate estimation of cell diameter d, intracellular volume fraction v_{in}, and water exchange rate constant k_{in} simultaneously.

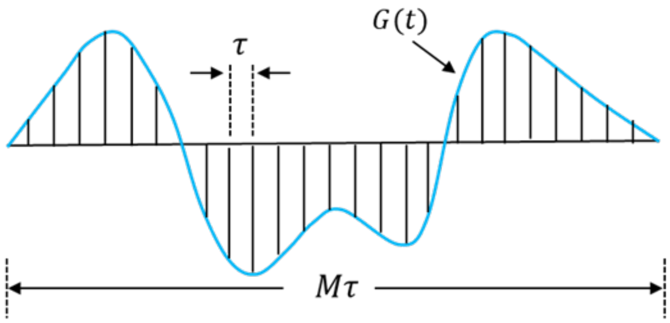

SUPPLEMENTARY FIGURE 2. Discretization of arbitrary waveform.
